# Intestinal Parasites and Hematological Parameters in Children Living in Ambatoboeny District, Madagascar

**DOI:** 10.3390/pathogens13110930

**Published:** 2024-10-25

**Authors:** Wanesa Richert, Daria Kołodziej, Danuta Zarudzka, Daniel Kasprowicz, Dariusz Świetlik, Krzysztof Korzeniewski

**Affiliations:** 1 Department of Epidemiology and Tropical Medicine, Military Institute of Medicine—National Research Institute, 04-141 Warsaw, Poland; wrichert@wim.mil.pl (W.R.); dkolodziej@wim.mil.pl (D.K.); dzarudzka@wim.mil.pl (D.Z.); 2 Clinique Medicale Beyzym, Ambatoboeny District, Manerinerina 403, Madagascar; daniel.kasprowicz@icloud.com; 3 Department of Biostatistics and Neural Networks, Medical University of Gdańsk, 80-210 Gdańsk, Poland; dswietlik@gumed.edu.pl

**Keywords:** STH, anemia, children, Madagascar

## Abstract

Madagascar is one of the poorest countries in the world. The country’s extreme weather conditions, poor sanitation, and weak economy facilitate the spread of parasitic diseases. Infections with intestinal parasites are particularly dangerous for children because they can cause malnutrition and anemia, which, in turn, have a negative effect on children’s cognitive functions and physical development. The aim of the present study was to analyze the prevalence of intestinal parasites and to assess hematological parameters in a group of children living in northern Madagascar. The screening was conducted in May 2024 in the Clinique Medicale Beyzym in Manerinerina, the Ambatoboeny district. It involved a sample of 208 children aged 0–17 years. Single stool samples were collected from all study participants. The samples were fixed in SAF fixative and then transported from Africa to Europe for further diagnostics at the Department of Epidemiology and Tropical Medicine, the Military Institute of Medicine–the National Research Institute in Poland. First, the samples were analyzed by light microscopy methods using three different diagnostic techniques (direct smear, decantation with distilled water, and the Fülleborn method). Next, they were tested by molecular biology methods (real-time PCR). Blood samples for the assessment of hematological parameters were collected at the healthcare center in Madagascar. The prevalence of intestinal parasites in the study sample was 61.5%. Helminths were found in 15.2% of the investigated children, and *Giardia intestinalis* (20.5%) was found to be the most prevalent parasite in the study population. Most infections were caused by potentially pathogenic stramenopila *Blastocystis* spp. (32.0%). Mean Hb, HCT, MCV, MCH, and MCHC levels in the study participants were below normal values. However, no correlation was found between the presence of a parasitic infection and low hematological parameters, which are a clinical sign of anemia. High rates of infections with intestinal parasites in children living in northern Madagascar support the necessity to introduce long-term preventive measures, which would limit the spread of parasitic diseases in the Malagasy population. Low hematological parameters in non-infected children may be indicative of persistent malnutrition or infection with other parasites, e.g., malaria or schistosomiasis.

## 1. Introduction

Madagascar is one of the poorest countries in the world, with over 80% of its population living in extreme poverty (with a daily income of USD 2.15). Poor personal hygiene, a lack of health education, and malnutrition facilitate the spread of intestinal parasitic infections (IPIs) in the country. The level of parasitism is directly correlated with low socio-economic factors, extreme climate conditions, and limited access to safe drinking water. Madagascar is the fourth-largest island in the world, located in the Indian Ocean, approximately 400 km off the eastern coast of Africa. The country experiences catastrophic cyclones several times a year. Heavy rains that hit Madagascar cause severe floods and the contamination of water tanks, which are the sources of drinking water for the local people. Sewage disposal and waste management are a challenge in Madagascar. The situation is further exacerbated by the common practice of open defecation [1,2]. Feces-contaminated soil is the source of soil-transmitted helminth (STH) infections, while contaminated drinking water is the source of *Giardia intestinalis*, *Entamoeba* spp., and *Cryptosporidium* spp. infections [3,4]. Although STH and protozoan infections can be asymptomatic in some cases, a large proportion of patients complain of symptoms such as persistent diarrhea, vomiting, and abdominal pain. Small children are at a particularly high risk of IPI transmission because of their poor hygiene practices. IPI infections that affect children are often associated with serious complications such as malnutrition and stunted growth. In addition, parasitic infestations of the gastrointestinal tract can cause serious blood loss due to hematophagous habits of hookworm or mechanical damage of the epithelium, which is caused by the disruption of the intestinal epithelial and endothelial barrier at the site of penetration or intestinal perforation caused by *Ascaris lumbricoides*. The above-mentioned pathologies result in the malabsorption of nutrients that leads to nutritional deficiencies, protein–energy malnutrition, and anemia [5,6]. The aim of the present study was to determine the prevalence and distribution of IPIs and to assess hematological parameters in children living in the Ambatoboeny district in northern Madagascar.

## 2. Materials and Methods

### 2.1. Study Population and Sample Collection

**Figure 1:**
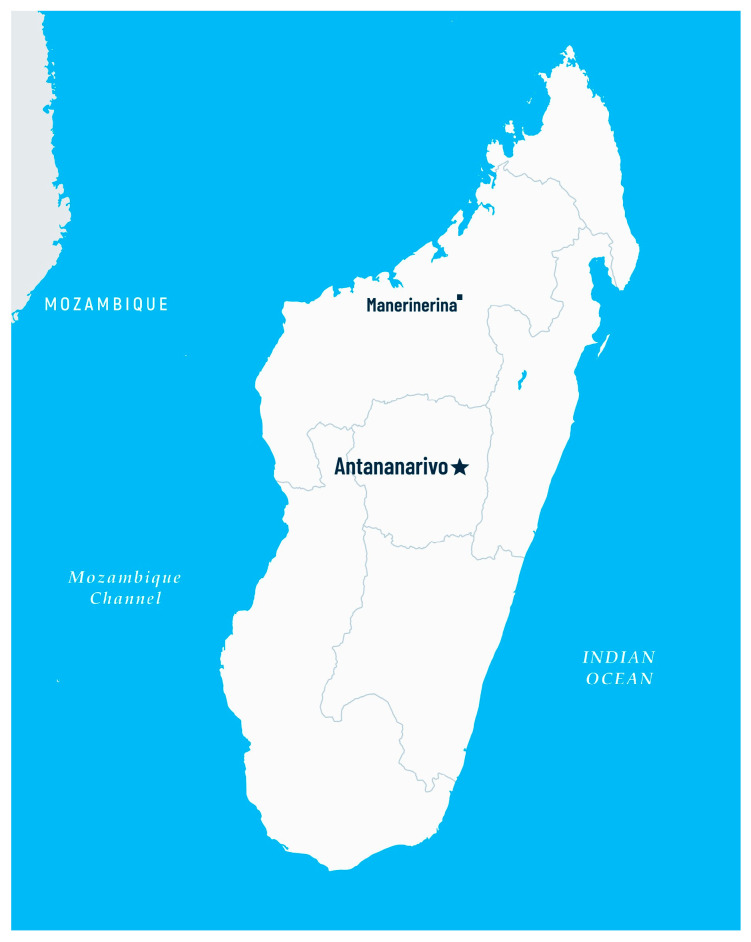
Map of Madagascar with location of Manerinerina, Ambatoboeny district (star marking–capital of the country, dot marking–small town where the research was conducted).

Screening for the prevalence of intestinal parasites and the assessment of hematological parameters was carried out in May 2024 in the Clinique Medicale Beyzym in Manerinerina, the Ambatoboeny district, in northern Madagascar (16°17′ S 47°17′ E; Figure 1). Biological material for parasitological diagnostics (stool samples) and venous blood samples for the assessment of hematological parameters were collected during the period of transition from the dry to the wet season (the wet season in northern Madagascar is from November to April). This study involved a group of 208 children aged between 0 and 17 years old. Each child could participate in the screening regardless of their health condition after informed consent was obtained from their parents/guardians. Information for the patients and the informed consent forms were translated into the Malagasy language. Venous blood samples were collected at the Clinique Medicale Beyzym by qualified personnel.

### 2.2. Data Collection

#### 2.2.1. Light Microscopy

The biological material for coprological diagnostics consisted of single stool samples provided by the study subjects. The samples were delivered to the Clinique Medicale Beyzym in Manerinerina on the day of collection. Next, the material was fixed in SAF fixative (sodium acetate–acetic acid–formalin), and two weeks later, it was transported to the Department of Epidemiology and Tropical Medicine of the Military Institute of Medicine–National Research Institute in Poland, where laboratory examination by light microscopy using three different diagnostic methods (direct smear in Lugol’s solution, decantation with distilled water, and the Fülleborn method) was performed [7].

#### 2.2.2. Molecular Investigation of the Fecal Samples

##### Pretreatment Before DNA Extraction

Before DNA extraction, washing steps were applied to the stool samples as a pretreatment, and the fixed stool samples (100–250 mg) were put into new clean test tubes and rinsed five times with distilled water to remove the SAF fixative. During the pre-analytic stage, each sample was centrifuged at 14,000 RPM for 5 min. Next, the supernatant was decanted, and 1 mL of distilled water was added. The sample was centrifuged at 14,000 RPM for 3 min. Next, the supernatant was decanted once again. The procedure was repeated four times, and the washed samples were stored at −20 °C until the next day.

##### Extraction of DNA from Fecal Samples

The Bosphore Nucleic Acid Extraction Versatile Spin Kit (Anatolia Geneworks, Istanbul, Turkey) was used to extract DNA from the samples. The kit works on the principle of using silica membrane spin columns to separate nucleic acid molecules. Before the extraction stage, the material was incubated in LTX reagent and proteinase K for 60 min at 50 °C. The next steps of the protocol were performed in line with the manufacturer’s instructions. The final eluate volume was 150 µL. The extracted samples were stored at −20 °C for further analysis.

##### DNA Amplification

The microscopic examination of the stool samples was expanded by molecular investigation to check the quality of the SAF-fixed feces samples, which were to be used for IPI detection. A commercial Bosphore Parasitic GI Panel Kit v1 (Anatolia Geneworks, Turkey) for real-time PCR in vitro diagnostics was used for the molecular diagnosis of intestinal parasites. The kit was used to detect and differentiate between *Ascaris* spp. (ITS-1 gene), *Taenia* sp. (ITS1 and 5.8S-rRNA genes), *Enterobius vermicularis* (COX1 gene), *Entamoeba histolytica* (5.8S rRNAITS2), *Cryptosporydium* spp. (18S-rRNA), and *Giardia intestinalis* (18S-rRNA). The amplifications were performed with an initial denaturation step (5 min at 95 °C), followed by 40 cycles of denaturation (15 s at 97 °C), the annealing of primers (1 min at 60 °C), and maintenance (2 min at 32 °C) in an AriaMx Real-Time PCR system (Agilent Technologies Inc., Santa Clara, CA, USA). The thermal conditions were identical for all the parasites tested. The entire protocol was conducted in line with the manufacturer’s instructions.

### 2.3. Hemoglobin Measurements

The hemoglobin concentration was measured in venous blood samples using the Mindray auto hematology analyzer BC-3000 Plus. The definition of anemia was based on the WHO criteria, where a hemoglobin level of ≥12.0 g/dL was regarded as normal, 11.0–11.9 g/dL as mild, 8.0–10.9 g/dL as moderate, and <8.0 g/dL as severe anemia.

### 2.4. Statistical Analysis

All statistical analyses were conducted using the TIBCO Software Inc., Palo Alto, CA, USA (2017) Statistica, version 13 (data analysis software system), available at http://www.statsoft.pl, URL (accessed on 8 September 2024). Chi-squared tests were applied to assess the independence of qualitative variables. Differences between two groups were evaluated using either Student’s *t*-test or the Mann–Whitney U test. A *p*-value of 0.05 was considered the threshold for statistical significance in all analyses.

### 2.5. Ethical Approval

This research project was approved by the Ministry of Public Health of Madagascar in Antananarivo (no. 108-24/MSANP/SPC of 5 April 2024). Parental consent was obtained for each child to participate in this study. The collection of samples was supervised by the medical personnel employed at the Clinique Medicale Beyzym from Marinerina (Ambatoboeny district, northern Madagascar).

### 2.6. Study Variables

The correlation between intestinal parasitic infections, gender, and age was examined. As STHs are one of the most important causes of anemia in children [6], the purpose of this study was to assess whether the occurrence of IPIs was associated with a lower hemoglobin (Hb) concentration and lower levels of other hematological parameters, which are a clinical sign of anemia.

## 3. Results

**Figure 2:**
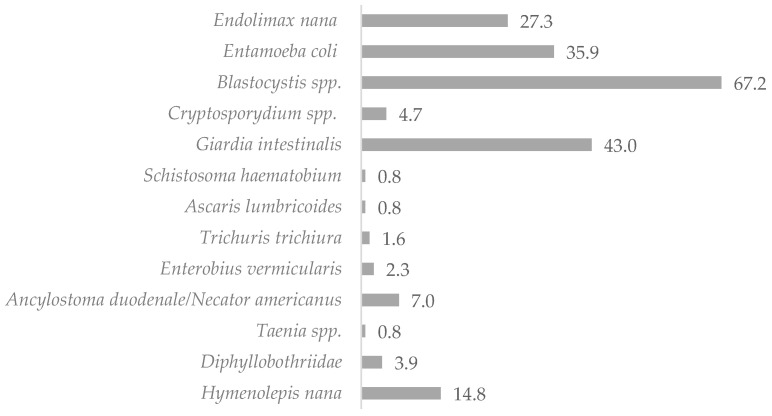
Percentage of infected children living in northern Madagascar in 2024 (n = 128).

The mean age of the study subjects was 11.8 years. Females accounted for 49.5% of the study group (n = 103), while males represented 50.5% of the sample (n = 105). Microscopic examination of the stool samples collected from the 208 children showed that 120 of the studied children (57.7%) were infected with intestinal parasites (non-pathogenic protozoan infections were not included). Molecular tests (RT-PCR) confirmed the results of the investigations by light microscopy methods and detected eight additional infections with pathogenic protozoa (including six *Cryptosporidium* spp. infections, whose detection would not have been possible with the microscopic methods used in this study, and two *Giardia intestinalis* infections), which determined the total prevalence of IPIs as 61.5%. Potentially pathogenic stramenopila *Blastocystis* spp. (32.0%) and pathogenic protozoa *Giardia intestinalis* (20.5%) were found to be responsible for most infections in the samples. Helminthic infections accounted for 15.2% of all infestations, of which 9.3% were caused by Cestodes, 5.6% by Nematodes, and 0.4% by Trematodes. The microscopic examination of the stool samples identified infections with 12 different pathogenic and non-pathogenic intestinal parasites, of which 30.1% were non-pathogenic *Entamoeba coli* and *Endolimax nana* protozoa. The distribution of IPIs in the children studied is shown in Table 1 and Figure 2.

**Figure 3:**
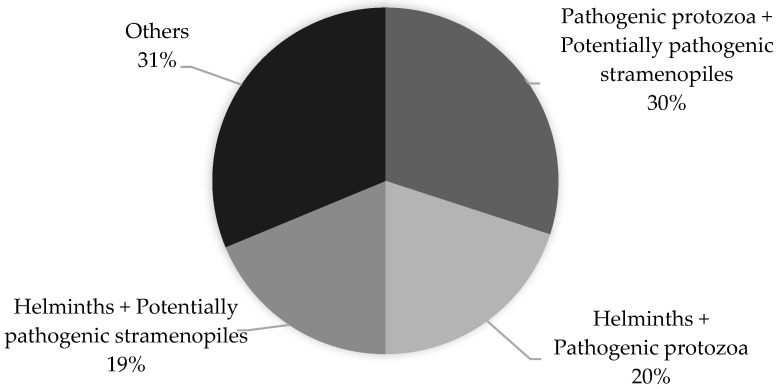
Polyparasitism in children living in northern Madagascar in 2024 (n = 208).

A total of 80 children involved in this study were found to be infected with more than one parasite (polyparasitism). The co-infections of *Blastocystis* spp. with pathogenic protozoa (*Giardia intestinalis* and *Cryptosporidium* spp.) and *Blastocystis* spp. with non-pathogenic protozoa were found to be the most prevalent (30% and 26%, respectively). The percentage distribution of the most commonly occurring co-infections is shown in Figure 3.

The female vs. male ratio was 1.0. The distribution of intestinal parasites detected in the Malagasy children was assessed by gender (female 64.1% vs. male 59.1%). Statistical analysis demonstrated that infections with pathogenic intestinal parasites (helminths + protozoa) were most often found in females (45.6% vs. 35.2%), while potentially pathogenic *Blastocystis* spp. were most often found in males (54.3% vs. 43.7%). Most IPIs were observed in the group of children aged 5–9 years old (71.6%), in which *Blastocystis* spp. (49.3%) infections prevailed. In all other age groups, the prevalence of IPIs ranged between 55 and 60%. Most cases of helminthiases were seen in adolescents aged 15–17 years and children aged 5–9 years (27.3% and 25.4%, respectively). We observed a significant reduction in the number of cases caused by pathogenic protozoa (*Giardia intestinalis* and *Cryptosporidium* spp.) with increasing age of the children and a considerable increase in the number of *Blastocystis* spp. infestations in children over 5 years old (40%); in contrast, in the group aged 0–4 years old, the rate of *Blastocystis* spp. infestations was 17.9%. Polyparasitism was seen in all age groups, with the lowest rates in children aged 0–4 years (28.6%) and the highest rates in children aged 5–9 years (43.28%). The distribution of intestinal pathogens in the individual study groups is shown in Table 2.

More than half of the children involved in this study had hemoglobin (Hb) concentrations below 12g/dL, of whom three children had Hb concentrations below 8 g/dL. The mean Hb concentration in the study group was 11.2 g/dL; in patients with IPIs, the mean Hb concentration was 11.3 g/dL. No correlation between infection with intestinal parasites and a drop in the Hb concentration was observed. The mean Hb concentration in children with helminthic infections was 11.4 g/dL; in children infected with pathogenic protozoa, it was 11.2 g/dL; and in non-infected children, it was 11.1 g/dL. Moreover, no correlation was found between the Hb concentration and sex. In total, 41.5% of females and 45.5% of males had low Hb concentrations (<11 g/dL). We observed, however, that the proportion of children with a Hb concentration of <12 g/dL decreased with increasing age. Most cases with low Hb concentrations were observed in the group aged 0–4 years (39.0%), while the fewest cases were observed in the group aged 15–17 years old (3.9%). The mean RBC count was slightly lower in patients with IPIs compared with non-infected individuals (4.5 × 10^6^/μL vs. 4.7 × 10^6^/μL), similar to the HCT count (35.8% vs. 36.3%) and the MCHC count (30.6 g/dL vs. 30.7 d/dL). The mean MCV and MCH counts were slightly lower in non-infected individuals (77.6 f/L vs. 79.5 f/L and 23.8 pg. vs. 24.7 pg., respectively) (Table 3). No correlation was found between the presence of an IPI and the values of hematological parameters, which are assessed in the diagnosis of anemia. We observed, however, that the mean HCT, MCV, MCH, and MCHC counts in all study participants were below the threshold values, which may be indicative of chronic malnutrition and/or an infection with other parasitic species.

## 4. Discussion

There are few up-to-date reviews examining the prevalence of IPIs in the Malagasy population. Due to the considerable geographical and climatic diversity of the island, as well as cross-country differences in sanitation access and practices, the prevalence of IPIs varies significantly depending on the region. A study by Greigert et al. [8] on the prevalence and distribution of intestinal parasites in residents of the Mahajanga town in northern Madagascar demonstrated high rates of protozoan infections in their sample, which is consistent with the results of the present study. They found that 72.8% of the study participants (n = 265) were infected with potentially pathogenic stramenopila or with pathogenic protozoa, of which *Blastocystis* spp. was predominant, similar to the present study. By analyzing the latest reports on the prevalence of IPIs in Madagascar and reviewing the results of the present study, we concluded that *Giardia intestinalis* [8,9] is the most prevalent pathogen in the country. It is worth noting, however, that the prevalence of *Giardia intestinalis* varies by region, with the highest prevalence seen in areas that are routinely flooded or located in the vicinity of canals and rivers [9]. The prevalence rate of *G. intestinalis* in the present study was found to be 20.5%, which was higher than in similar studies conducted in other parts of Madagascar (7.9%) [8], Rwanda (10.9%) [10], and Ethiopia (6.2%) [11], but similar to the rates demonstrated in a previous study by the authors of this article that was carried out on a comparable sample of children in 2023 (21.2%) [12]. The proportion of *Cryptosporidium* spp. infections was similar to the one found in the north-western parts of Madagascar (2.9% and 2.6%, respectively [8]) but significantly lower than in a study conducted in Gabon (19%) [13]. Other studies conducted among African populations (including the Malagasy people) reported the same protozoan infections as in this study, as well as infections caused by *Dientamoeba fragilis*, *Balantidium coli,* and microsporidia [8,13]. None of these parasites was detected in this study; however, this might have been the result of the limitations of this study and the diagnostic techniques applied. The present study showed a high prevalence rate of IPIs (61.5%) in Madagascar, which is consistent with the findings of other authors: 77% [8], 96.3% [5], 71.4% [14], and are characteristic values for other African countries, e.g., Ethiopia (83.8%) [11], Côte d’Ivoire (55.2%) [15], Rwanda (53.2%) [10], and Gabon (61%) [13]. The prevalence of helminthiases in the present study was higher than in cases studied at the Gastroenterology Department in Mahajanga (7.9%) [8] but lower than in residents of the villages surrounding the Ranomafana National Park, where >70% of individuals were infected with *Ascaris lumbricoides* or *Trichiuris trichura*, and 33% had a hookworm infection [14,16]. The prevalence of STHs varies cross-country, with most cases observed along the eastern coast of the island [17]. A high prevalence of non-pathogenic protozoa and potentially pathogenic stramenopila reflects poor sanitation access and practices in the region; their presence indicates widespread fecal contamination of water and soil in the region [18]. The study findings give evidence that poor sanitation is a significant issue in Manerinerina, northern Madagascar. STH and protozoa infections are often associated with anemia. IPIs affect all age groups, but children are most susceptible to infection because their immune system is not yet fully developed, and their immune response is weaker compared with adults [19,20]. Our study found that *Hymenolepis nana* was the most common helminthic infection in the study participants. In fact, *Hymenolepis nana* infections are widespread in Africa, which is supported by the results of the studies by Greigert et al. [8] and Razafiarimanga et al. [9]. *Hymenolepis nana* infections are considered a serious threat to public health because, apart from being transmitted via contaminated food and water, they can also be transmitted from person to person, which can lead to local outbreaks, and they can cause autoinfection. Furthermore, *H. nana* eggs can be found in water used for irrigating crops, which could potentially become yet another common mode of *H. nana* transmission [21]. The prevalence rate of *H. nana* infections in this study was found to be 9.1%, which was higher than in other studies conducted in Madagascar (0.4–2.5%) [8,9,22] but lower than in other African countries [13,21]. The present study found hookworm infections (*Ancylostama duodenale/Necator americanus*) in 3.4%, *Trichiuris trichiura* infections in 0.7%, and *Ascaris lumbricoides* in 0.4% of the samples. As a comparison, a study by Greigert et al. [8] that was carried out in the northwestern parts of Madagascar found that the prevalence rates of the above-listed parasites were 1.1%, 1.1%, and 1.5%, respectively. The results obtained were lower than those reported in other African countries [10,11,13,15]. The mean hemoglobin concentration in patients infected with these parasites was 11.7 g/dL. Of all soil-transmitted helminths responsible for causing anemia, hookworm is the most important. The parasite produces anticoagulants, which prevent blood clotting but also digest the host’s erythrocytes, causing blood loss. It has been estimated that an adult *N. americanus* or *A. duodenale* nematode can ingest from 0.05 to 0.2 mL of blood per day, causing an average daily blood loss of 26.4 mL in infected adults [23]. The blood loss is more severe in high-intensity infections [24]. *T. trichiura* and *A. lumbricoides* infections can also be associated with anemia. *T. trichiura* can cause chronic bleeding from the cecum or the colon at the site of the parasite’s (its adult forms) penetration. The average daily blood loss per single *T. trichiura* is 0.005 mL, which means that in the case of more intensive invasions, the blood loss can be more severe, and it may lead to anemia. In nematode infections, anemia can develop as a result of intestinal obstruction and perforation of the ileum, while in chronic ascariasis, it is associated with malnutrition [25]. Helminthic infections detected in the study participants were either mild or moderate and, therefore, were not directly responsible for anemia in the infected patients. This study found a single case of a severe *Taenia* sp. infection. According to the literature, *Taenia* sp. invasions are highly endemic in Madagascar [26]. The infected child had a Hb concentration of only 6.9 g/dL. Although tapeworm infections are not currently considered to be directly responsible for iron deficiency anemia [27], there are case reports that suggest there is a correlation between *Taenia* sp. infection and anemia [28,29,30,31]. The present study found five cases of *Diphyllobothriidae* invasion. Diphyllobothriidae species that can infect humans include *Diphyllobothrium latum*, *D. nihonkaiense*, *D. dendriticum*, *D.stemmacephalum*, *D. balaenopera*, and *D. pacificum*. The intermediate hosts of these parasites are freshwater and marine fish (most often perch, burbot, pike, and salmon). This infestation is most commonly transmitted through the consumption of contaminated raw fish. The most widespread species of *Diphyllobothriidae* is *D. latum*, but *D. stemmacephalum* and *D. balaenopterea* are also considered cosmopolitan. *Diphyllobothriidae* may be transferred to non-endemic areas through the trade of wild fish [32]. In his study “Infectious Diseases of Madagascar”, Berger [33] stated the occurrence of *D. latum* in Madagascar. There is little information in the scientific literature about the occurrence of other species of *Diphyllobothriidae*, but the above report may suggest that the detected cases of infection are caused by *D. latum*. To confirm this, additional diagnostics should be performed. Infections by *Diphyllobothriidae* usually cause vitamin B_12_ deficits, but they are rarely associated with anemia [34]. When analyzing the link between IPIs and anemia, one cannot forget that Madagascar is an extremely poor country where poor sanitation is of particular concern. For this reason, we cannot assume that intestinal parasitic infections are the only factor responsible for the high anemia prevalence in the local people. Madagascar is severely affected by malnutrition and other serious public health issues other than IPIs. For example, schistosomiasis is prevalent in more than 90% of the country’s districts, and malaria is endemic across the whole territory of Madagascar (with the exception of mountainous regions) [17,35]. A study by Adewale et al. [36] demonstrated that individuals infected with *Schistosoma* spp. were two times more likely to develop anemia compared with non-infected individuals. Our study found one case of *Schistosoma mansoni* infection; the infected individual had a hemoglobin concentration of 7.6 g/dL. Although no correlation between the presence of IPIs and anemia was found in the present study, attention was drawn to the low hematological parameters of the children participating in this study (Hb, MCHC, MCH, MCV, and HCT). The highest prevalence of anemia was observed in children aged 0–4 years (the mean Hb concentration increased with the increasing age of children), which was most likely attributable to the accelerated growth and expansion of blood volume in the first months of life [37]. Madagascar is predominantly an agricultural country, with the main crops including rice and cassava, i.e., plants that require irrigation. The water used for irrigation by local farmers is often contaminated and, therefore, of questionable quality and epidemiological status. Livestock farming is predominantly concentrated on the production of cattle and pigs, but poor food safety practices and a lack of public health veterinary activities encourage the spread of *Taenia* sp. infections in local settings. Fishing is another important sector of agriculture in Madagascar; however, as is the case with livestock farming, neither fishing nor fish handling is regulated by the law, and the consumption of contaminated fish carries the risk of infection by *Diphylobotriidae* tapeworms [1]. Additionally, the proximity of the ocean and fishing poses a risk of infection with other fish-borne parasites, such as *Anisakis simplex* and *Pseudoterranova decipiens* [33]. Cases of anemia reported in Madagascar are largely the effect of poverty and malnutrition, being the result of poor-quality diets and nutritional deficiencies (a lack of essential microelements). In addition, Madagascar is not only endemic for IPIs but for other infectious diseases as well, including malaria and schistosomiasis, which both carry a higher risk of developing anemia than IPIs. A lack of correlation between anemia and IPIs in Malagasy children was well documented in previous studies [12,38]. The findings suggest the necessity to extend future studies to other factors that contribute to the development of anemia, especially in the pediatric population.

## 5. Conclusions

A high prevalence of IPIs in the group of Malagasy children involved in this study reflects the poor sanitation access and practices in the local community. A considerable proportion of the children investigated had anemia, a condition that can potentially lead to stunted growth and learning difficulties in the future. Further research into the prevalence of IPIs, as well as other parasitic diseases that may be responsible for causing anemia (e.g., schistosomiasis or malaria), is needed. It is, likewise, important to examine environmental and socio-cultural risk factors potentially responsible for nutritional deficits in the local communities. Another important issue that needs consideration is ensuring drinking water safety. In this respect, it is necessary to provide parents with specialist advice on child nutrition. To effectively control and prevent the spread of IPIs in the region, it is necessary to conduct routine parasitological screening and, if necessary, implement mass deworming campaigns.

## 6. Limitations of the Study

The authors used molecular tests (real-time PCR) to examine fecal samples fixed in SAF fixative. Ögren et al. [39] developed a method for DNA recovery from SAF-fixed fecal samples and compared the real-time PCR detection of protozoa from SAF-fixed and unpreserved fecal samples. They found that the detection rate in SAF-fixed samples was equal to the detection rate in unpreserved samples. Also, Lee et al. [40] successfully extracted *Giardia intestinalis* DNA from formalin-fixed samples. They observed, however, that PCR amplification was limited to short fragments of DNA. There are reports that suggest that formaldehyde fixation of biological samples impedes DNA extraction due to cross-linking between nucleic acids and DNA fragmentation. The time between fixation and lysis/extraction of DNA plays a key role in the recovery of genetic material from samples [41]. Therefore, the time of exposure of the sample to the fixative should be limited, and lysis for DNA extraction should be performed as quickly as possible.

## Figures and Tables

**Table 1:** Frequencies of intestinal parasites in children from northern Madagascar in 2024 (n = 208).

Intestinal Parasite	Number of Infections (Percentage of Tested Children; n = 208); Light Microscopy	Number of Infections (Percentage of Tested Children; n = 208); Real-Time PCR	Number of Infections (Percentage of Tested Children; n = 208); Both Methods
Total number	(64.4)	(22.6)	(67.8)
Helminths	41 (19.7)	1 (0.5)	41 (19.7)
Cestodes	25 (12.0)	1 (0.5)	25 (12.0)
*Hymenolepis nana*	19 (9.1)	n.a.	19 (9.1)
Diphyllobothriidae	5 (2.4)	n.a.	5 (2.4)
*Taenia* sp.	1 (0.5)	1 (0.5)	1 (0.5)
Nematodes	15 (7.2)	0	15 (7.2)
*Ancylostoma duodenale * *Necator americanus*	9 (4.3)	n.a.	9 (4.3)
*Enterobius vermicularis*	3 (1.4)	0	3 (1.4)
*Trichuris trichiura*	2 (1.0)	n.a.	2 (1.0)
*Ascaris lumbricoides*	1 (1.5)	n.a.	1 (0.5)
Trematodes	1 (0.5)	n.a.	1 (0.5)
*Schistosoma mansoni*	1 (0.5)	n.a.	1 (0.5)
Pathogenic protozoa	43 (20.7)	48 (23.1)	61 (29.3)
*Giardia intestinalis*	43 (20.7)	42 (20.2)	55 (26.4)
*Cryptosporydium* spp.	0 (0.0)	6 (2.9)	6 (2.9)
Potentially pathogenic stramenopila	86 (41.4)	n.a.	86 (41.4)
*Blastocystis* spp.	86 (41.4)	n.a.	86 (41.4)
Non-pathogenic protozoa	81 (39.0)	n.a.	81 (39.0)
*Entamoeba coli*	46 (22.2)	n.a.	46 (22.2)
*Endolimax nana*	35 (16.8)	n.a.	35 (16.8)

n.a. (not applicable)—these parasites were not a part of the PCR panel in the used diagnostic kit.

**Table 2:** Distribution of intestinal parasites in children by sex and age in northern Madagascar in 2024 (n = 208).

	Sex		Age			
	F	M	0–4	5–9	10–14	15–17
No. of tested children	103 (49.5)	105 (50.5)	56 (26.9)	67 (32.2)	63 (30.3)	22 (10.6)
Positive (+)	66 (64.1)	62 (59.1)	31 (55.4)	48 (71.6)	36 (57.1)	13 (59.1)
Helminths	21 (20.4)	20 (19.1)	8 (14.3)	17 (25.4)	11 (17.5)	6 (27.3)
Cestodes	14 (13.6)	11 (10.5)	4 (7.1)	10 (14.9)	7 (11.1)	5 (22.7)
Nematodes	7 (6.8)	8 (7.6)	3 (5.4)	7 (10.5)	4 (6.3)	1 (4.6)
Trematodes	0 (0.0)	1 (1.0)	1 (1.8)	0 (0.0)	0 (0.0)	0 (0.0)
Pathogenic protozoa	26 (25.2)	17 (16.2)	22 (39.3)	21 (31.3)	14 (22.2)	2 (9.1)
*Blastocystis* spp.	45 (43.7)	57 (54.3)	10 (17.9)	33 (49.3)	26 (41.3)	9 (40.9)
Co-invasions	45 (60.8)	35 (53.1)	16 (28.6)	29 (43.3)	20 (31.7)	9 (40.1)

**Table 3:** Hematological parameters in infected vs. non-infected children in northern Madagascar in 2024 (n = 208).

Variables	Parasitic Infections (−) (n = 80)	Parasitic Infections (+) (n = 128)	Total (n = 208)	*p*-Value
Age (years)				0.5787 ^1^
Mean (SD)	7.9 (4.8)	8.3 (4.3)	8.1 (4.5)	
Range	1.0–17.0	1.0–17.0	1.0–17.0	
Median	8.0 (8.0)	9.0 (6.0)	8.0 (7.0)	
95% CI	[6.8; 8.9]	[7.5; 9.0]	[7.5; 8.7]	
Sex				0.4560 ^3^
Female	37 (46.3%)	66 (51.6%)	103 (49.5%)	
Male	43 (53.7%)	62 (48.4%)	105 (50.5%)	
Body weight (kg)				0.7474 ^1^
Mean (SD)	23.0 (12.3)	22.3 (11.2)	22.5 (11.6)	
Range	5.0–53.0	4.0–54.0	4.0–54.0	
Median	23.5 (16.0)	20.0 (13.0)	21.0 (15.0)	
95%CI	[20.2; 25.7]	[20.3; 24.2]	[21.0; 24.1]	
Hb g/dL				0.2178 ^1^
Mean (SD)	11.0 (1.2)	11.3 (1.3)	11.2 (1.3)	
Range	7.6–13.4	6.9–15.5	6.9–15.5	
Median	11.2 (1.8)	11.5 (1.5)	11.4 (1.5)	
95%CI	[10.8; 11.3]	[11.0; 11.5]	[11.0; 11.4]	
RBC				0.0140 ^1^
Mean (SD)	4.7 (0.4)	4.5 (0.5)	4.6 (0.5)	
Range	3.6–6.1	2.5–5.6	2.5–6.1	
Median	4.7 (0.5)	4.5 (0.5)	4.6 (0.6)	
95%CI	[4.6; 4.8]	[4.4; 4.6]	[4.5; 4.6]	
HCT				0.9279 ^2^
Mean (SD)	35.9 (3.4)	36.0 (4.0)	36.0 (3.8)	
Range	25.3–41.6	22.4–50.5	22.4–50.5	
Median	35.8 (4.5)	36.3 (4.8)	36.3 (4.8)	
95% CI	[35.2; 36.7]	[35.3; 36.7]	[35.4; 36.5]	
MCHC				0.0343 ^1^
Mean (SD)	30.7 (1.3)	30.9 (2.0)	30.8 (1.8)	
Range	26.1–33.1	19.0–33.2	19.0–33.2	
Median	30.8 (2.2)	31.2 (1.8)	31.0 (1.9)	
95% CI	[30.4; 31.0]	[30.5; 31.2]	[30.6; 31.1]	
MCH				0.0047 ^2^
Mean (SD)	23.7 (2.9)	24.8 (2.5)	24.4 (2.7)	
Range	15.4–29.0	18.0–30.9	15.4–30.9	
Median	24.0 (3.5)	25.2 (3.0)	24.6 (3.0)	
95% CI	[23.1; 24.4]	[24.4; 25.3]	[24.0; 24.8]	
MCV				0.0169 ^2^
Mean (SD)	77.4 (8.1)	80.0 (7.4)	79.0 (7.8)	
Range	51.4–93.7	63.2–99.6	51.4–99.6	
Median	78.1 (10.7)	79.9 (9.0)	78.9 (9.6)	
95% CI	[75.6; 79.2]	[78.7; 81.3]	[77.9; 80.0]	

^1^ Mann–Whitney U test; ^2^ Student’s *t*-test; ^3^ Chi-square test.

## Data Availability

The data presented in this study are available on request from the corresponding author.
